# Development and Validation of Broad-Range Qualitative and Clade-Specific Quantitative Molecular Probes for Assessing Mercury Methylation in the Environment

**DOI:** 10.1128/AEM.01271-16

**Published:** 2016-09-16

**Authors:** Geoff A. Christensen, Ann M. Wymore, Andrew J. King, Mircea Podar, Richard A. Hurt, Eugenio U. Santillan, Ally Soren, Craig C. Brandt, Steven D. Brown, Anthony V. Palumbo, Judy D. Wall, Cynthia C. Gilmour, Dwayne A. Elias

**Affiliations:** a Biosciences Division, Oak Ridge National Laboratory, Oak Ridge, Tennessee, USA; b Smithsonian Environmental Research Center, Edgewater, Maryland, USA; c University of Missouri, Columbia, Missouri, USA; University of Calgary

## Abstract

Two genes, *hgcA* and *hgcB*, are essential for microbial mercury (Hg) methylation. Detection and estimation of their abundance, in conjunction with Hg concentration, bioavailability, and biogeochemistry, are critical in determining potential hot spots of methylmercury (MeHg) generation in at-risk environments. We developed broad-range degenerate PCR primers spanning known *hgcAB* genes to determine the presence of both genes in diverse environments. These primers were tested against an extensive set of pure cultures with published genomes, including 13 Deltaproteobacteria, nine Firmicutes, and nine methanogenic Archaea genomes. A distinct PCR product at the expected size was confirmed for all *hgcAB*^+^ strains tested via Sanger sequencing. Additionally, we developed clade-specific degenerate quantitative PCR (qPCR) primers that targeted *hgcA* for each of the three dominant Hg-methylating clades. The clade-specific qPCR primers amplified *hgcA* from 64%, 88%, and 86% of tested pure cultures of Deltaproteobacteria, Firmicutes, and Archaea, respectively, and were highly specific for each clade. Amplification efficiencies and detection limits were quantified for each organism. Primer sensitivity varied among species based on sequence conservation. Finally, to begin to evaluate the utility of our primer sets in nature, we tested *hgcA* and *hgcAB* recovery from pure cultures spiked into sand and soil. These novel quantitative molecular tools designed in this study will allow for more accurate identification and quantification of the individual Hg-methylating groups of microorganisms in the environment. The resulting data will be essential in developing accurate and robust predictive models of Hg methylation potential, ideally integrating the geochemistry of Hg methylation to the microbiology and genetics of *hgcAB*.

**IMPORTANCE** The neurotoxin methylmercury (MeHg) poses a serious risk to human health. MeHg production in nature is associated with anaerobic microorganisms. The recent discovery of the Hg-methylating gene pair, *hgcA* and *hgcB*, has allowed us to design and optimize molecular probes against these genes within the genomic DNA for microorganisms known to methylate Hg. The protocols designed in this study allow for both qualitative and quantitative assessments of pure-culture or environmental samples. With these protocols in hand, we can begin to study the distribution of Hg-methylating organisms in nature via a cultivation-independent strategy.

## INTRODUCTION

The trace metal mercury (Hg) is a widespread global pollutant with a >4-fold increase in atmospheric emissions since antiquity (1). While all forms of Hg are toxic, the neurotoxin monomethyl Hg (MeHg) poses the most health risks due to its toxicity and biomagnification in food webs (2). MeHg production in nature is predominantly microbial (3) and is limited to strictly anaerobic microorganisms (4, 5). Methylation has commonly been tied to microbial sulfate reduction (6–8), but only a subset of sulfate-reducing microbes are capable of Hg methylation (4, 9). Hg methylation has also been linked to methanogenesis (10, 11) and Fe(III) reduction (12, 13) in particular locations. However, within these latter groups, only Geobacter spp. and hydrogenotrophic or methylotrophic Methanomicrobia species contain the necessary genes (4). Hence, the relative importance of individual strains, or even of groups of microbes, in MeHg production in nature remains poorly constrained.

The recent discovery of the first genes, *hgcA* and *hgcB*, linked to Hg methylation provides the foundation for evaluating the distribution of Hg-methylating microorganisms in nature. The conserved gene pair (*hgcAB*) was identified by comparative genomics and shown by genetics to be essential for converting Hg(II) to MeHg (14). Every tested Hg-methylating species has the gene pair (*hgcAB*^+^), which is absent (or heavily altered) in all nonmethylators (4, 14). These observations suggest that *hgcAB* distribution could be used to predict the distribution of Hg methylators in nature and that measurement of the genes and their expression could be used to predict the potential for Hg methylation. Several primer sets have been designed to screen for *hgcA* alone or the *hgcAB* gene pair using PCR-based amplification and have been tested in temperate soils (15), rice paddy soils (16), wetlands (15, 17), and chloralkali plant runoff (18). In each case, primer sets were designed based on genomic DNA (gDNA) *hgc* sequences from a small subset of the known Hg-methylating microorganisms, most commonly Deltaproteobacteria. Perhaps because these primers amplify *hgcA* or *hgcAB* from only a subset of Hg methylators, there has been limited success to date in linking Hg methylator distribution to environmental processes. The advent of more efficient and comprehensive molecular probes for Hg methylation genes will provide a first look at the distribution of these organisms among environments. Further development of molecular approaches for gene expression should provide an important improvement upon current tools for measuring Hg methylation potential (e.g., Hg isotope tracer studies [19]) that are slow, are costly, and provide no information on the organisms involved in the process.

Here, we detail the development and validation of primer sets to detect and quantify *hgcAB*^*+*^ organisms in the environment based upon the full set of currently available Hg methylators with sequenced genomes. We developed a broad-range degenerate PCR primer set that spanned both genes (*hgcA* and *hgcB*). The *hgcAB* primer set was designed to provide the most comprehensive possible coverage and confirmation of *hgcAB*^*+*^ species, since both genes are required for biological Hg methylation (14). We used 84 *hgcAB* genomes and 31 reference strains (more than double the numbers used in previous studies) to obtain a more complete representation of *hgcAB* diversity (4) and limited PCR primer degeneracy for improved accuracy. We also developed quantitative PCR (qPCR) primers to separately quantify *hgcA*^*+*^ in Deltaproteobacteria, Firmicutes and Archaea. Due to qPCR limitations and the lack of conserved sequence areas, *hgcAB* qPCR was not possible, so we targeted the *hgcA* active site and highly conserved adjacent regions. Finally, to begin to evaluate the utility of our primer sets, we tested *hgcA* and *hgcAB* recovery from sand and sediment amended with *hgcAB*^+^ pure cultures.

## MATERIALS AND METHODS

### Pure culture strains, culturing, media, and sample collection.

Thirty-one strains with published genomes (13 Deltaproteobacteria, 9 Firmicutes, and 9 methanogenic Archaea) were used in primer development and testing and assayed for the presence and abundance of an *hgc*-specific amplicon. All microorganisms either had *hgcAB* in their genome or were closely related to the Hg-methylating strains and were selected as negative controls (see Table S1 in the supplemental material). Strains were obtained from the Deutsche Sammlung von Mikroorganismen und Zellkulturen (https://www.dsmz.de) culture collection and other sources (see Table S1 in the supplemental material). Cultures were grown anaerobically in the media and conditions listed. Additional modifications to growth conditions are described in the text in the supplemental material for select cultures: Desulfovibrio inopinatus (Dv. inopinatus), Ethanoligens harbinense, Methanospirillum hungatei, Methanofollis liminatans, Methanocorpusculum bavaricum, Methanosphaerula palustris E1-9c, and Methanocella paludicola SANAE. Culture stocks were stored in 10% (vol/vol) glycerol at −80°C, and purity was checked by 16S rRNA sequencing. DNA was isolated from fresh cultures and frozen cell pellets.

Pure culture gDNA was extracted with a Wizard genomic DNA purification kit (Promega, Madison, WI) according to the manufacturer's Gram-positive protocol or with the PowerLyzer Powersoil kit (MoBio, Carlsbad, CA) with a TissueLyser (Qiagen, Valencia, CA) for the cell disruption step. Typically, samples were obtained from 1 × 10^8^ to 1 × 10^9^ pelleted stationary-phase cells (fresh or frozen). The pellets were washed twice with equal volumes of 50 mM Tris-HCl (pH 8). Final DNA concentrations for PCR/qPCR method development were measured with Qubit (Thermo Fisher Scientific, Waltham, MA) (see Table S1 in the supplemental material). Nanodrop ND-1000 (Thermo Fisher Scientific) measurements of *A*_260_/*A*_280_ and *A*_260_/*A*_230_ ratios were used to assess purity (see Table S1 in the supplemental material).

### Development and testing of a broad-range qualitative *hgcAB* primer set.

**FIG 1:**
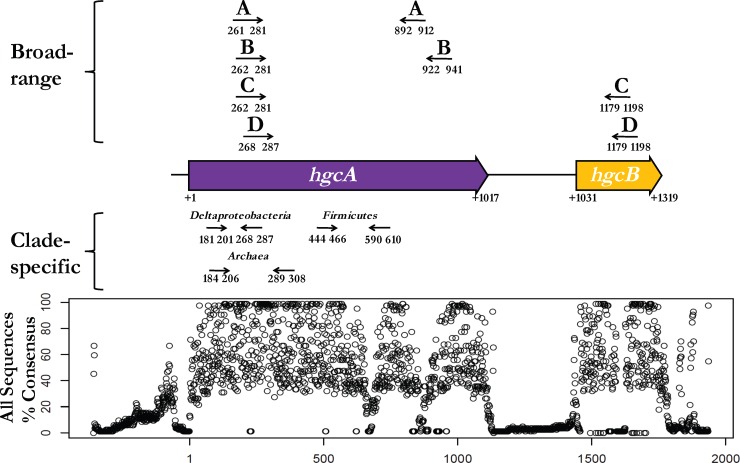
Schematic representation of primer locations within *hgcAB* relative to Dv. desulfuricans ND132. Sequence consensus at each base position among ≥80 *hgcAB*^+^ microorganisms, including Deltaproteobacteria, Firmicutes, and Archaea. Position 1 corresponds to the GTG putative start codon for *hgcAB*. Arrows indicate primer positions for the broad-range *hgcAB* qualitative and the clade-specific *hgcA* quantitative PCR pairs. Base pair positions indicated for A (15), B (16), and C (17) in the figure are the locations of the forward and reverse primers from previous studies. D refers to the location of the broad-range *hgcAB* primer set designed in this study. The clade-specific qPCR primers designed in this study are annotated by their respective clade.

Several degenerate primers were designed and tested (see Table S2 in the supplemental material). The putative *hgcAB* gene sequences (identified in 84 strains, including 47 Deltaproteobacteria, 22 Firmicutes, 13 Archaea, and 2 currently unclassified strains) were retrieved from the National Center for Biotechnology Information (http://www.ncbi.nlm.nih.gov) and aligned with MUSCLE (20). The initial alignment was manually refined in AliView v1.17 (21) (Fig. 1) and separately with Clustal X2 (22). The forward primer primer-binding region was positioned across the highly conserved *hgcA* cap-helix motif. The reverse primer was positioned across the highly conserved *hgcB* ferredoxin-type iron-sulfur binding domain. Primers were designed to amplify an ∼950-bp region across *hgcA* and *hgcB* and are illustrated in Fig. 1. Final primer degeneracy was kept below 48-fold to limit nonspecific amplification or heterologous dimerization (Table 1). Oligonucleotides were purchased from Integrated DNA Technologies (Coralville, IA) or Eurofins Genomics (Huntsville, AL).

**TABLE 1:** List of final primers

Primer name	Sequence^*c*^	Start^*a*^ (5′) (bp)	Stop^*a*^ (3′) (bp)	Length (bp)	Primer melting temp (^o^C)	Degeneracy (fold)	Amplicon length (bp)	Primer concn (nM)	Avg detection limit (copies)
ORNL-HgcAB-uni-F	5′-AAYGTCTGGTGYGCNGCVGG-3′	268	287	20	58.1–68.3	48	818–1,020^*b*^	1,000	1 × 10^6^
ORNL-HgcAB-uni-R	5′-CABGCNCCRCAYTCCATRCA-3′	167	148	20	57.3–66.0	96		1,000	
ORNL-Delta-HgcA-F	5′-GCCAACTACAAGMTGASCTWC-3′	181	201	21	52.9–56.3	8	107	250	2 × 10^5^
ORNL-Delta-HgcA-R	5′-CCSGCNGCRCACCAGACRTT-3′	287	268	20	60.7–68.3	32		250	
ORNL-SRB-Firm-HgcA-F	5′-TGGDCCGGTDARAGCWAARGATA-3′	444	466	23	53.6–62.5	72	167	250	2 × 10^5^
ORNL-SRB-Firm-HgcA-R	5′-AAAAGAGHAYBCCAAAAATCA-3′	610	590	21	45.5–53.8	18		250	
ORNL-Archaea-HgcA-F	5′-AAYTAYWCNCTSAGYTTYGAYGC-3′	184	206	23	48.9–61.6	512	125	500	2 × 10^4^
ORNL-Archaea-HgcA-R	5′-TCDGTCCCRAABGTSCCYTT-3′	308	289	20	54.0–64.7	72		250	
ORNL-D-ND132-F	5′-GCCAACTACAAGCTGACCTTC-3′	181	201	21	56.0	1	107	250	1 × 10^2^
ORNL-D-ND132-R	5′-CCCGCCGCGCACCAGACGTT-3′	287	268	20	68.3	1		250	

a Start and stop position correspond to *hgcA* or *hgcB* nucleotide sequence of Dv. desulfuricans ND132. Only ORNL-HgcAB-uni-R is in *hgcB*.

b Typical amplicon length range for those strains examined and tested in this study.

c Y = C/T, N = A/T/C/G, V = A/C/G, B = C/G/T, R = A/G, M = A/C, S = C/G, W = A/T, D = A/G/T, H = A/C/T.

Prospective primers were tested iteratively on gDNA extracted from several control strains containing or lacking *hgcAB*. Combinations of up to 21 forward and 19 reverse primers were tested (see Table S2 in the supplemental material) with the goal of amplifying a single DNA band from *hgcAB*^+^ cultures without false positives. For optimization, multiple enzymes were tested; the two described in detail are platinum *Taq* DNA polymerase (Thermo Fisher Scientific) and Apex TaqRed (Genesee Scientific, San Diego, CA). Both of these enzymes worked well for this application in our hands. PCRs were set up according to the manufacturer's protocol and processed on a Mastercycler Pro (Eppendorf, Hauppauge, NY) or PTC-200 thermal cycler (MJ Research, St. Bruno, QC, Canada). Once selected, the final primer set and protocol were tested against all 31 microorganisms with the chosen reaction conditions. The final protocol is, in brief, a 20-μl reaction mixture prepared for each strain containing 1 × 10^6^ copies of gDNA, 1 μM each primer, and specified reagents provided with each enzyme. Lower gDNA copy numbers were also examined to determine detection limits (data not shown). A touchdown step was necessary to accommodate the range in melting temperatures for both the forward and reverse primers (58.1 to 68.3°C and 57.3 to 66.0°C, respectively) (see Tables S3 and S4), such that a single ∼950-bp product was observed and to avoid the production of a smeared product at 0 to 500 bp. Amplification was initiated with preincubation (2 min at 98°C), 5 cycles of denaturation (30 s at 98°C), annealing (30 s at 68°C; −1°C/cycle), and extension (30 s at 72°C), followed by 30 cycles of denaturation (30 s at 98°C), annealing (30 s at 63°C), and extension (60 s at 72°C) and a final incubation step (2 min at 72°C) (see Table S5 in the supplemental material). The initial 5 cycles facilitated gDNA binding with a greater GC content, i.e., Deltaproteobacteria, while limiting nonspecific amplification. The second, lower, annealing temperature allowed for gDNA priming from the remaining strains. Selection of the final primer set and protocol was based on iterative testing of pure cultures, described in more detail in the text in the supplemental material.

Amplification products were subjected to electrophoresis to check for a band at the expected size. For products amplified with platinum *Taq* DNA polymerase, 18 μl was mixed with 2 μl of 10× loading dye (Thermo Fisher Scientific) and subjected to electrophoresis (85 V, 120 min) in 1% (wt/vol) agarose gels. Products amplified with Apex TaqRed were added directly to the gel. To confirm the correctly sized (∼950 bp) PCR product was *hgcAB*, the gel band was isolated and purified with Wizard SV gel and the PCR clean-up system (Promega), Sanger sequenced with the degenerate primers for both strands at the University of Tennessee Molecular Biology Resource Facility, and aligned to known *hgcAB* sequences (data not shown).

### Development of quantitative clade-specific *hgcA* primer sets.

Quantitative clade-specific degenerate *hgcA* primer sets were designed for each of the three phylogenetic clades (Table 1). A lack of conserved regions in the sequences immediately upstream and downstream from the gene junction meant that it was not possible to design a primer set that would amplify across *hgcA* and *hgcB*. Additionally, the spacer between *hgcA* and *hgcB* varies in length among organisms, and, in rare cases, the gene pair is not adjacent along the chromosome. We chose a SYBR green-based approach rather than TaqMan due to limited conserved sequences within *hgcAB* to create an additional probe. These primer sets were designed in a similar manner as the broad-range *hgcAB* primers, except that the desired amplicon was 100 to 200 bp and the primer sequences were designed to maximize clade specificity. To provide clade selectivity and specificity, *hgcA* primers were designed within the conserved cap-helix and transmembrane regions and iteratively tested on gDNA from our 31 cultures. Combinations of typically up to 10 forward and 10 reverse primers were tested for each clade (see Table S2 in the supplemental material). For these primer sets, the goal was amplification of a single PCR product from the highest percentage of *hgcAB*^+^ cultures within each target clade, without false positives in *hgcAB*^+^ cultures from other clades, and without any PCR product from microorganisms lacking *hgcAB*. To limit false positives, either from nonspecific amplification or heterologous dimerization, our goal was to limit the degeneracy for each primer to 72-fold, but this was not possible in every case. The minimum information for publication of quantitative real-time PCR experiments guidelines, or the MIQE guidelines (23, 24), were followed during the design and optimization process.

Primer testing at Oak Ridge National Laboratory (ORNL) was done with iQ SYBR green supermix (Bio-Rad, Hercules, CA) according to the manufacturer's protocol on a C1000 Touch real-time PCR detection system. Data were analyzed with CFX Manager (version 3.1; Bio-Rad) by single-threshold analysis and/or with the curve fit to regression for comparison (25–27). Primer testing at the Smithsonian Environmental Research Center (SERC) was performed on a ViiA 7 real-time PCR system (Applied Biosystems) and analyzed with the associated software. For each strain and clade-specific primer set, a standard curve (seven 4-fold serial dilutions, 2.5 × 10^6^ to 1.5 × 10^2^ gDNA copies per reaction) was generated to calculate primer efficiency (*E* = 10^(−1/slope)^), where the slope of the line is derived from the standard curve and %*E* = (*E* − 1) × 100. In brief, 5 μl of specified gDNA (5 × 10^5^ to 3 × 10^1^ copies per μl) was loaded in triplicate to 12-tube strip tubes or 96-well low-profile plates and dried at 50°C for ∼30 min, followed by the addition of a 20-μl aliquot of clade-specific master mix. Bovine serum albumin (New England BioLabs, Inc., Ipswich, MA) was included in some reactions. For all qPCRs, the fluorescent signal was acquired after each extension step. The melt curve protocol included annealing at the extension temperature and melting at a ramp rate of 0.5°C/5 s up to 95°C, with the fluorescent signal acquired continuously during the melt curve. For each triplicate PCR, one sample was subjected to electrophoresis through a 3% (wt/vol) agarose gel (85 V, 90 min).

### Selected reaction conditions for each primer set.

The final and optimized amplification protocols for each clade are described in Table S5 in the supplemental material. For Deltaproteobacteria-specific qPCR, the amplification protocol was initiated with a 3-min preincubation at 95°C and 30 cycles of denaturation (15 s at 95°C) and annealing plus extension (20 s at 65°C). The final forward (ORNL-Delta-HgcA-F) and reverse (ORNL-Delta-HgcA-R) primer concentrations were each 250 nM. For Firmicutes-specific qPCR, the amplification protocol was initiated with a 3-min preincubation at 95°C and 30 cycles of denaturation (10 s at 95°C), annealing (10 s at 47°C), and extension (60 s at 58°C). The final forward (ORNL-SRB-Firm-HgcA-F) and reverse (ORNL-SRB-Firm-HgcA-R) primer concentrations were each 250 nM. For methanogenic Archaea-specific qPCR, the amplification protocol was initiated with a 3-min preincubation at 95°C and 30 cycles of denaturation (30 s at 95°C), annealing (10 s at 50°C), and extension (60 s at 55°C). The final forward (ORNL-Archaea-HgcA-F) and reverse (ORNL-Archaea-HgcA-R) primer concentrations were 500 nM and 250 nM, respectively. For the Archaea, the high degree of primer degeneracy due to poor *hgcAB* sequence conservation required a forward primer with 512-fold degeneracy but allowed for inclusion of most Archaea tested. However, nonspecific amplification was observed from non-Archaea gDNA templates after the 30-cycle cutoff.

### Environmental amendment experiment.

As a first assessment of primer efficacy and specificity in natural samples, the recovery of *hgcA* from *hgcAB*^+^ cultures spiked into clean sand and a natural sediment was evaluated. Both matrices were amended with known cell densities of one culture from each *hgcAB*^+^ clade. Fine sea-sand (Thermo Fisher Scientific) was added to Erlenmeyer flasks in 50-g aliquots prior to being autoclaved. Course-grain sediment was collected from East Fork Poplar Creek (EFPC) at the National Oceanographic and Atmospheric Administration site, designation EFK 22.3, at Oak Ridge National Laboratories, TN (28). The stream is shallow and moderately fast flowing, and deep anaerobic sediments are uncommon. Mercury is the major contaminant and exists predominantly in highly sequestered forms (i.e., organically complexed mercury), with total sediment Hg levels of ∼20 to 50 mg/kg (28). Fifty grams of fresh creek bottom sediment were collected by hand, with a 50-ml conical tube, from an area of sediment accumulation among the generally cobbly bottom, under ∼1 m of water. Sediments were stored on ice until returned to the laboratory. The sediment was dried on a heat block, and 2-g aliquots were measured and placed in grinding vials (SPEX Sample Prep, Metuchen, NJ). Since the sediment was known to undergo Hg methylation, we expected that *hgcAB*^+^ microorganisms would be identified, while the clean sterilized sand was used as a negative control with limited numbers of microorganisms.

The sand and EFPC sediment were spiked with 1 × 10^8^, 1 × 10^7^ or 1 × 10^6^ cells per dry gram sediment from stationary-phase Desulfovibrio desulfuricans (Dv. desulfuricans) ND132 (Deltaproteobacteria), Desulfococcus metallireducens (Firmicutes), and Methanolobus tindarius (Ml. tindarius) (Archaea) and stored overnight at −80°C to emulate environmental sample collection and storage. Cell counts for the culture spikes were determined by hemocytometer with an Axioskop 2 Plus microscope (Carl Zeiss Microscopy, Thornwood, NY). For the strains being tested, the clade-specific qPCR primer sets were further tested for sensitivity, i.e., 40 cycles compared to 30 cycles (see Table S6 in the supplemental material). The copy number of *hgcA* in the culture spikes was determined by extracting the gDNA from cells and quantifying using the clade-specific qPCR primers (see Table S7 in the supplemental material). DNA was extracted from sand and sediment using a modified version of the ORNL2012 protocol (29, 30). Typically, ∼1 μg of gDNA per gram of sediment was recovered from samples amended with 1 × 10^8^ cells, with an *A*_260_/*A*_280_ ratio close to 2.0. For all samples, extracted gDNA was diluted (1:10 and 1:100) and assayed for *hgcAB* presence and *hgcA* abundance with protocols established in this study.

## RESULTS AND DISCUSSION

### Broad-range qualitative protocol for *hgcAB* diversity.

**FIG 2:**
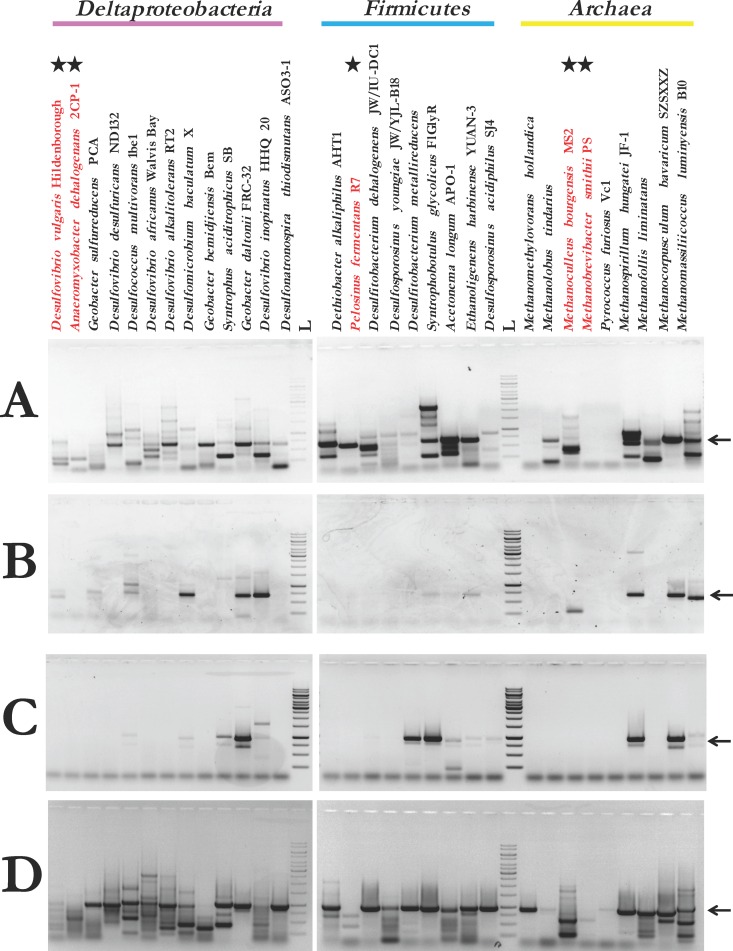
Qualitative broad-range PCR results among protocols. PCR with specified primer sets from previous studies (A, Schaefer et al., 2014; B, Liu et al., 2014; and C, Bae et al., 2014) (15–17), including the newly designed primers for this study (D). Organisms in red are starred and denote microorganisms that do not encode *hgcAB* and should not generate a product, while the rest are *hgcAB*^*+*^ microorganisms that should have a product. Arrow denotes expected band size position (∼950 bp). L, 1 kb plus ladder from Thermo Fisher Scientific.

Refinement of the annealing temperatures, extension times, and polymerase selection resulted in significantly improved PCR-based detection of *hgcAB* from pure cultures (Fig. 2) and was consistent between the ORNL and SERC laboratories (see Table S3 in the supplemental material). For initial experiments, an annealing temperature gradient (55 to 70°C) was used to cover a wide temperature range (see Tables S3 and S4). As expected, strains with greater GC content required a higher annealing temperature for amplification. For most Deltaproteobacteria, >64°C improved *hgcAB* amplification and reduced nonspecific amplification. The Firmicutes showed optimal amplification at 59°C, while the Archaea had a broader GC content percentage range, so the temperatures were more variable. Overall, an annealing temperature of 63°C accommodated most strains (see Fig. S1 in the supplemental material).

The Invitrogen platinum *Taq* DNA polymerase and Apex TaqRed were tested and compared, and differences were noted (see Fig. S1 in the supplemental material). We predicted that the use of an enzyme that was not engineered to be less promiscuous, Apex TaqRed, might better amplify strains whose sequences deviated from the primer sequences and, therefore, serve to amplify more environmental genomes for *hgcAB*. However, because both enzymes worked well on gDNA isolated from pure culture, we do not endorse one enzyme over the other. Because multiple bands were obtained from each pure culture PCR, it is advisable to separate the products for >2 h by electrophoresis and gel purify the band before sequencing.

The final primer set and protocol properly identified 94% (29 of 31) of the tested strains with a band at ∼950 bp for Hg methylators, and sequencing verified *hgcAB* as the PCR product (Fig. 2; see also Table S1 in the supplemental material). This is a substantial improvement from previous reports, since, in our hands, the protocol used by Schaefer et al. properly identified 84% (15) while those of Liu et al. (16) and Bae et al. (17) identified 45% and 61% of these microorganisms, respectively (Fig. 2). The only *hgcAB*^+^ Deltaproteobacteria not detected properly by our protocol was Dv. inopinatus; however, direct PCR amplification was not feasible, because *hgcA* and *hgcB* are separated by many genes (http://www.ncbi.nlm.nih.gov/assembly/GCF_000429305.1). A weak product also appeared for the Desulfovibrio vulgaris strain lacking *hgcAB*. Sequencing revealed that the product was not homologous to *hgcAB* but instead had multiple off-target products. Accurate detection of the Deltaproteobacteria is critical, since this clade contains the highest number of methylators, the sulfate-reducing bacteria (SRB) which have long been implicated as important methylators, and Geobacter spp. which are arguably the strongest methylators (4). Hence, the Deltaproteobacteria should be a large portion of the *hgcAB* community in sulfate-reducing environments, such as the Florida Everglades. Previous work by Bae et al. (2014) (17) showed this to be the case and that there were also some Firmicutes and methanogenic Archaea. Bae et al. further concluded that syntrophic Deltaproteobacteria are the major source of Hg methylation in the Florida Everglades. However, use of their protocol in our hands with pure strains of Hg methylators showed that limited diversity may have been captured, including 4/11 Deltaproteobacteria, 5/8 Firmicutes, and 2/7 Archaea (Fig. 2C). These results, along with the recent finding that specific algorithms should be used to filter out false positives from the protein family in which HgcAB resides (5) and that only 8 of the known 78 Hg-methylating Deltaproteobacteria reside in the order Syntrophobacterales (see http://www.esd.ornl.gov/programs/rsfa/data/PredictedMethylators/PredictedMethylators_20160420.pdf [from 25 April 2016]), suggest that the abundance of syntrophic Deltaproteobacteria may have been overestimated and the presence of nonsyntrophic sulfate-reducing Deltaproteobacteria may have been underestimated.

Several organisms that encode *hgcA* and *hgcB* in an unusual format, including fused and separated genes, were included in the study for comprehensiveness. Our protocol gave very weak amplification of *hgcAB* at 1 × 10^6^ copies gDNA from the archaeon Pyrococcus furiosus Vc 1, an organism that was amplified according to the protocol by Schaefer et al. (15). This hyperthermophile is divergent in *hgcAB* nucleotide sequence from other Hg methylators (31), contains a fused *hgcAB*, does not methylate at near-boiling temperatures (5), and has four additional mismatches against our primer sequences (see Table S3 in the supplemental material). While Desulfovibrio africanus (Dv. africanus) Walvis Bay (32) and Dv. inopinatus do possess *hgcA* and *hgcB*, the genes are separated. For Dv. africanus, one gene separates *hgcA* and *hgcB* for an expected band size of 2,365 bp. Amplification produced the expected 2,365-bp amplicon as well as a separate ∼950-bp band. Cloning and sequencing revealed that the latter was a phosphoribosyl pyrophosphate amidotransferase (desaf_3140-1) (data not shown). For Dv. inopinatus, *hgcA* and *hgcB* are on opposites sides of the chromosome, and PCR by our protocol was not possible, as the product size was too large. Last, for the strong methylator Geobacter bemidjiensis Bem (4), the expected product was produced, although poorly, along with other smaller products. Reasons for this are currently unclear, as there is only one mismatch between the degenerate primers and the sequence.

### Clade-specific qPCR primers.

Quantitative *hgcA* primer sets were developed and optimized for each of the three clades. For the Deltaproteobacteria and Archaea primers, the reverse primers were positioned within the conserved cap-helix, a GC-rich region, and the forward primers were sited in another conserved region about 100 to 125 bp upstream. After testing, we were able to slightly offset the primer landing pads for the two clades within these regions based on specific areas of conservation for each (Fig. 1). The final Deltaproteobacteria primers had 0 to 3 mismatches with tested Deltaproteobacteria hgcA sequences and >3 mismatches for *hgcA* in the other clades. For the strains tested, the overall primer amplification efficiencies ranged from ∼60 to 90%, varying with the sequence match and with temperature for the landing pads in each organism (see Tables S3 and S4 in the supplemental material). Estimated detection limits ranged from roughly 10^3^ to 10^5^ *hgcA* copies. Under these conditions, qPCR yielded a single product, as determined by melt curve analysis (data not shown) and gel electrophoresis (see Fig. S2), for 7 of the 11 *hgcAB*^*+*^ Deltaproteobacteria (Table 2). Four Deltaproteobacteria generated false negatives (i.e., Dv. africanus, Syntrophus aciditrophicus SB, Dv. inopinatus, and Desulfonatronospira thiodismutans [Dn. thiodismutans] ASO3-1) at 2.5 × 10^6^ gDNA copies. This limitation was likely due to divergent gene sequences (see Table S3). With one exception, the chosen Deltaproteobacteria qPCR primers did not amplify a product from any species tested in other clades or from two Deltaproteobacteria lacking *hgcAB* (see Fig. S2).

**TABLE 2:** Results of clade-specific qPCR of *hgcA*

Genome source	Template	*hgcA* within genome^*a*^	Deltaproteobacteria qPCR primer set		Firmicutes qPCR primer set		Methanogenic Archaea qPCR primer set	
			qPCR efficiency (%)	Detection limit^*b*^	qPCR efficiency (%)	Detection limit^*b*^	qPCR efficiency (%)	Detection limit^*b*^
Deltaproteobacteria	Dv. vulgaris Hildenborough DSM-644	−	N.A.^*c*^	B.D.^*d*^	N.A.	B.D.	N.A.	B.D.
	Anaeromyxobacter dehalogenans 2CP-1 DSM-21875	−	N.A.	B.D.	N.A.	B.D.	N.A.	B.D.
	Geobacter sulfurreducens PCA DSM-12127	+	79	2.4 × 10^3^	N.A.	B.D.	N.A.	B.D.
	Dv. desulfuricans ND132	+	72	9.8 × 10^3^	N.A.	B.D.	N.A.	B.D.
	Desulfococcus multivorans 1be1 DSM-2059	+	62	1.6 × 10^5^	N.A.	B.D.	N.A.	B.D.
	Dv. africanus Walvis Bay	+	N.A.	B.D.	N.A.	B.D.	N.A.	6.3 × 10^5^
	Desulfovibrio alkalitolerans RT2 DSM-16529	+	76	9.8 × 10^3^	N.A.	B.D.	N.A.	B.D.
	Desulfomicrobium baculatum X DSM-4028	+	82	6.3 × 10^5^	N.A.	B.D.	N.A.	2.5 × 10^6^
	Geobacter bemidjiensis Bem DSM-16622	+	N.A.	6.3 × 10^5^	N.A.	B.D.	N.A.	B.D.
	Syntrophus aciditrophicus SB DSM-26646	+	N.A.	B.D.	N.A.	B.D.	N.A.	6.3 × 10^5^
	Geobacter daltonii FRC-32 DSM-22248	+	87	9.8 × 10^3^	N.A.	B.D.	N.A.	B.D.
	Dv. inopinatus HHQ 20 DSM-10711	+	N.A.	B.D.	N.A.	1.6 × 10^5^	N.A.	B.D.
	Dn. thiodismutans ASO3-1 DSM-19093	+	N.A.	B.D.	N.A.	B.D.	N.A.	6.3 × 10^5^
Firmicutes	Dethiobacter alkaliphilus AHT 1 DSM-19026^*e*^	+	N.A.	B.D.	70	1.6 × 10^5^	N.A.	B.D.
	Pelosinus fermentans R7 DSM-17108	−	N.A.	B.D.	N.A.	B.D.	N.A.	B.D.
	Df. dehalogenans JW/IU-DC1 DSM-9161^*e*^	+	N.A.	B.D.	N.A.	6.3 × 10^5^	N.A.	B.D.
	Desulfosporosinus youngiae JW/YJL-B18 DSM-17734^*e*^	+	N.A.	B.D.	84	1.6 × 10^2^	N.A.	2.5 × 10^6^
	Df. metallireducens 853-15A DSM-15288^*e*^	+	N.A.	B.D.	88	1.6 × 10^2^	N.A.	B.D.
	Syntrophobotulus glycolicus FlGlyR^*f*^ DSM-8271	+	N.A.	B.D.	N.A.	2.5 × 10^6^	N.A.	B.D.
	Acetonema longumAPO-1 DSM-6540^*f*^	+	N.A.	B.D.	N.A.	2.5 × 10^6^	N.A.	B.D.
	Ethanoligenens harbinense YUAN-3 DSM-18485^*f*^	+	N.A.	B.D.	N.A.	B.D.	N.A.	B.D.
	Desulfosporosinus acidiphilus SJ4 DSM-22704^*e*^	+	N.A.	B.D.	75	3.9 × 10^4^	N.A.	B.D.
Archaea	Methanomethylovorans hollandica DMS1 DSM-15978	+	N.A.	B.D.	N.A.	B.D.	73	3.9 × 10^4^
	Ml. tindarius DSM-2278	+	N.A.	B.D.	N.A.	B.D.	79	2.9 × 10^4^
	Methanoculleus bourgensis MS2 DSM-3045	−	N.A.	2.5 × 10^6^	N.A.	B.D.	N.A.	B.D.
	Methanobrevibacter *smithii* PS DSM-861	−	N.A.	B.D.	N.A.	B.D.	N.A.	B.D.
	Pyrococcus furiosus Vc 1 DSM-3638	+	N.A.	B.D.	N.A.	B.D.	N.A.	B.D.
	Methanospirillum hungatei JF-1 DSM-864	+	N.A.	B.D.	N.A.	2.5 × 10^6^	102	1.3 × 10^4^
	Methanofollis liminatans DSM-4140	+	N.A.	B.D.	N.A.	B.D.	62	3.9 × 10^3^
	Methanocorpusculum bavaricum SZSXXZ DSM-4179	+	N.A.	B.D.	N.A.	B.D.	87	4.9 × 10^4^
	Methanomassiliicoccus luminyensis B10 DSM-25720	+	N.A.	B.D.	N.A.	B.D.	74	3.7 × 10^4^
	Methanosphaerula palustris E1-9c^*g*^	+	N.T.^*h*^	N.T.	N.T.	N.T.	N.T.	N.T.
	Methanocella paludicola SANAE^*g*^	+	N.T.	N.T.	N.T.	N.T.	76	1.3 × 10^3^

a +, present in genome; −, absent in genome.

b Lowest number of copies detected.

c N.A., not applicable.

d B.D., below detection limit at 30 cycles.

e Sulfate-, sulfite-, thiosulfate-, and S^0^-reducing Firmicutes.

f *hgcAB*^+^ non-SRB Firmicutes. The Firmicutes-specific qPCR protocol was designed to exclude these strains, and there should be no or limited amplification observed.

g Strain that was tested by our protocol but not part of the complete study.

h N.T., not tested.

The relatively low amplification efficiencies for these primers were caused, at least in part, by primer degeneracy and primer mismatches with some species. To evaluate the impact of primer degeneracy, a Dv. desulfuricans ND132 specific primer set was tested (Table 1; see also Fig. S3 in the supplemental material) using the same base locations and protocol. The Deltaproteobacteria degenerate primer pair contained one set of sequences that was a perfect match for *hgcA* in strain ND132. The nondegenerate primer pair amplified Dv. desulfuricans ND132 gDNA with 98% efficiency, compared to 70% for the degenerate primer set. Lower efficiencies underestimate copy numbers. For example, 6.25 × 10^5^ gDNA copies yielded a critical threshold cycle (*C*_T_) of 18.6 ± 0.2 using the degenerate primer set, compared to 12.4 ± 0.1 for the nondegenerate primer pair. This difference of 6.2 cycles would underestimate the total copies by ∼75-fold.

The methanogenic Archaea qPCR primers were located slightly downstream of the Deltaproteobacteria primers (Fig. 1). Sequence divergence in methanogen *hgcA* is high, so a highly degenerate primer pair was needed to capture that diversity. The calculated forward and reverse primer temperatures were 54.0 to 66.0°C and 54.0 to 64.0°C, respectively; therefore, we were constrained to 50.0 to 55.0°C annealing and extension temperatures. Lower annealing temperatures were examined; however, increased nonspecific amplification was observed (data not shown). The chosen pair amplified a single product of the correct size from 6 of the *hgcAB*^+^ methanogens tested (Table 2), as determined by melt curve analysis (data not shown) and gel electrophoresis (see Fig. S4 in the supplemental material). Amplification efficiencies ranged from ∼62 to 102%, and estimated detection limits ranged from roughly 10^3^ to 5 × 10^4^ *hgcA* copies (Table 2; see also Table S3). For those Archaea that amplified best, typically fewer than 2 mismatches were observed against the primer sequences (see Table S4). No amplification was observed for methanogens lacking *hgcAB* or from Pyrococcus furiosus, in which the *hgcA* and *hgcB* genes are fused (5). Because of the high degeneracy (512-fold) for the Archaea forward primer, nonspecific amplification was observed for many non-Archaea strains, but, fortunately, products were not typically observed until after cycle 30. Exceptions were amplification at cycle ∼25 for Syntrophus aciditrophicus with an incorrect band size and Dn. thiodismutans with the correct band size, as observed by melt curve analysis and gel electrophoresis (see Fig. S4).

For the Firmicutes qPCR primer set, we were unable to locate the annealing sites in the cap-helix region of the gene because of insufficient sequence specificity for Firmicutes in that region. A workable primer set was located further downstream in regions that were conserved among the most *hgcAB*^+^ Firmicutes (Fig. 1). Firmicutes hgcA sequences are widely divergent, potentially a result of multiple horizontal gene transfers into the group (5). To produce a reasonable level of primer degeneracy, we focused primer development on sulfate-, sulfite-, and thiosulfate-reducing (here designated SRB) Firmicutes, the most evolutionarily similar *hgcA* gene grouping within the clade (see Table S4 in the supplemental material). For the final primer set, the SRB Firmicutes strains tested in culture had 0 to 3 mismatches (see Tables S3 and S4) with calculated forward and reverse temperatures of 53.6 to 62.5°C and 45.5 to 53.8°C, respectively. The exceptionally low primer temperature was due to the low GC content for Firmicutes *hgcA* genes. We found it important to follow a 3-step qPCR protocol that included a 47°C annealing step to allow all primers to bind and a separate 58°C extension step to allow for optimal polymerase activity (see Table S5).

The Firmicutes qPCR primer set was tested on all 31 gDNAs (Table 2). A single product was observed for all SRB Firmicutes strains, with amplification efficiencies ranging from 70 to 90% and estimated detection limits ranging from roughly 10^2^ to 10^6^ *hgcA* copies. No product was produced for non-SRB Firmicutes or non-Firmicutes strains, as determined by melt curve analysis (data not shown) and gel electrophoresis (see Fig. S5 in the supplemental material). As expected, Dethiobacter alkaliphilus AHT-1 showed the poorest efficiency (see Table S3 in the supplemental material), since 12 of the 44 primer bases were mismatches. Desulfitobacterium dehalogenans (Df. dehalogenans) did not amplify even though there were only 3 mismatches; the low temperature (44.1°C) for the reverse primer may be the cause. False positives were generated from Dv. inopinatus and Methanospirillum hungatei JF-1, but only at the highest abundance tested (2.5 × 10^6^ copies). Interestingly, for Dv. inopinatus, 19 of the 44 bases were mismatches, suggesting that the product amplified was not *hgcA*, although the product was never gel purified and sequenced.

To summarize, we developed qPCR primers that are highly inclusive of known Hg-methylating organisms and highly clade specific. Average amplification efficiencies for *hgcA* using these degenerate primers were roughly 80% for each of the 3 major clades of Hg methylation microbes, and detection limits averaged between about 10^3^ to 10^4^ *hgcA* copies for most organisms tested. Efficiencies varied between 60 and 100% among individual organisms, probably driven by differences in degeneracy, by the degree of match to the primers, and by differences in temperature. These differences in qPCR primer response among organisms will need to be considered as the research community begins method development for application of these primers in nature. Another limitation of the degenerate primer PCR approach is relatively poor detection limits (>10^5^ copies of *hgcA*) for some organisms, particularly Firmicutes that do not reduce sulfur. Detection limits are of particular concern in environments where overall cell densities are low, or where Hg-methylating organisms make up a small fraction of the community. Table S3 in the supplemental material gives qPCR efficiency and detection limits for many of the organisms used for primer development.

### Sediment proof-of-principle experiment.

As a first test of the primers in complex natural matrices, we evaluated the recovery of *hgcA* and *hgcAB* from Hg-methylating organisms spiked into either sterile sand or freshwater sediment. EFPC creek sediment and autoclaved sand were each spiked with equal cell densities of the 3 cultures, Dv. desulfuricans ND132, Desulfitobacterium metallireducens (Df. metallireducens) and Ml. tindarius, ranging from 10^6^ to 10^8^ cells per gram of dry weight of material. Genomic DNA was extracted from cultures and spiked samples.

**FIG 3:**
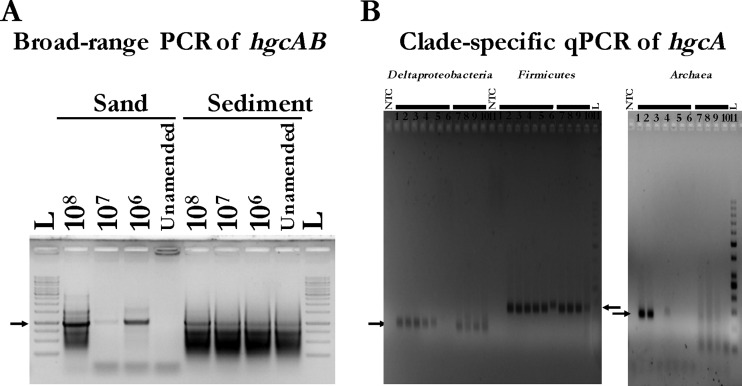
*hgcAB*^+^ microorganism spiking experiment. (A) Broad-range PCR results of *hgcAB* with gDNA isolated from autoclaved sand and sediment samples spiked with specified cell copies per dry gram sample with each clade-specific strain: Dv. desulfuricans ND132, Df. metallireducens and Ml. tindarius. L, 1 kb plus ladder from Thermo Fisher Scientific. Arrow points to the 1-kb band. Expected product size is ∼950 bp. (B) Clade-specific qPCR results of *hgcA* with isolated gDNA from spiked sediment samples, with specified cell copy number for each representative strain, 1 × 10^8^, 1 × 10^7^, 1 × 10^6^, and unamended per dry gram sediment (lanes 8 to 11, respectively). Standard curve with gDNA from a representative strain (Dv. desulfuricans ND132, Df. metallireducens, and Ml. tindarius) from each clade (Deltaproteobacteria, Firmicutes, and Archaea, respectively) for each clade-specific protocol at 1 × 10^7^, 1 × 10^6^, 1 × 10^5^, 1 × 10^4^, 1 × 10^3^, 1 × 10^2^, and 1 × 10^1^ cell copies per qPCR (lanes 1 to 7, respectively). L, low-range O′GeneRuler DNA ladder from Thermo Fisher Scientific. Arrow denotes the expected product size for each amplicon (Deltaproteobacteria, 107 bp; Firmicutes, 167 bp; Archaea, 125 bp).

The universal PCR primer set yielded a single product, of the expected size, from gDNA from both spiked samples (Fig. 3), along with smaller nonspecific amplified products of ∼0 to 750 bp. No band was observed in the unamended autoclaved sand, but, as expected, gDNA from the unspiked EFPC sediment yielded a presumptive *hgcAB* band. We found that the 5-cycle annealing touchdown step was required to amplify *hgcAB* from the EFPC gDNA samples, while nonspecific amplification products were produced with or without touchdown. The inclusion of a touchdown step should be evaluated during method development for future environmental samples. Interestingly, it appeared that the sand sample spiked with 10^7^ cells did not amplify as well as when spiked with 10^6^ cells. After analysis of all samples by qPCR (discussed below; see also Table S7 in the supplemental material), it was clear that the gDNA from the sand sample spiked with 10^7^ cells was extracted poorly, at about 20% of theoretical levels, compared with 50% and 40% for 10^8^ and 10^6^ cells, respectively.

To evaluate the efficacy of the quantitative primers, we directly compared qPCR results for gDNA extracted from individual organisms with gDNA recovered from the sand and sediment samples, assuming gDNA extraction efficiency would be similar for comparison. To evaluate the *hgcA* amplification from the individual organisms, qPCR was performed on 10-fold dilutions of gDNA, from 10^7^ to 10^1^ genome copies per reaction (see Table S6 in the supplemental material). Forty amplification cycles were run to test detection limits. For the specific organisms used, the qPCR primers were most sensitive for the Df. metallireducens strain and least sensitive for Ml. tindarius. Using gDNA from 10^6^ cells per reaction, qPCRs yielded *C*_T_s of 16, 21, and 28, respectively, for Df. metallireducens, Dv. desulfuricans ND132, and Ml. tindarius (see Table S6). Detection limits, based on the number of copies detected at 30 cycles, were roughly 10^2^, 5 × 10^3^, and 5 × 10^5^, respectively, for these organisms. Lower *hgcA* copy numbers were detectable with more cycles; however, environmental samples should probably be limited to 30 cycles because of nonspecific amplification from some primers at high cycle numbers.

**FIG 4:**
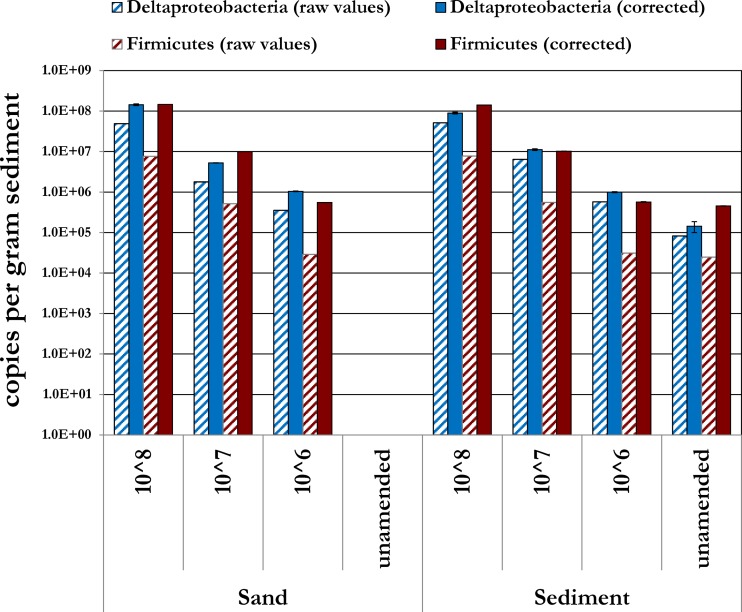
Recovery of *hgcA* from spiked sand and sediment using the clade-specific qPCR primers. Sand and sediment were spiked separately with Dv. desulfuricans ND132 (Deltaproteobacteria), Df. metallireducens (Firmicutes), and Ml. tindarius (Archaea) at the specified cell density per gram of dry weight. Bars show recovered raw abundance of *hgcA* copies and corrected copy numbers after accounting for extraction and amplification efficiency for each clade. No copies of *hgcA* were detected for Deltaproteobacteria or Firmicutes for the unamended sand sample. Archaea data are not shown, as no discrete amplicon of the correct length was detected in the samples.

Amplification of gDNA extracted from the spiked sediment and sand samples with the clade-specific qPCR primers produced amplicons of the proper size for *hgcA* in Deltaproteobacteria and Firmicutes (Fig. 3). The overall efficiency of *hgcA* recovery from the spiked samples was determined directly for each of the clades by comparing the copy number of *hgcA* in DNA extracted from the spike sand and soil (minus unamended control values) to the *hgcA* copy number in DNA extracted from the cultures used as spikes (see Table S7 in the supplemental material). Here, we assumed that cells had one copy of *hgcA* per chromosome, based on genomic sequences of known methylators (5). For example, for sand amended with 1.0 × 10^8^ Dv. desulfuricans ND132 cells, ∼4.83 × 10^7^ cells were recovered, assuming one copy of *hgcA* per cell as determined by qPCR based on comparison with a Dv. desulfuricans ND132 gDNA standard curve. Therefore, a Deltaproteobacteria extraction efficiency of ∼48% was determined for sand amended with 1 × 10^8^ cells. All extraction efficiencies are shown in Table S7. The *hgcA* abundance from sediment was always higher due to endogenous organisms. The Firmicutes extraction efficiencies for sand and sediment were much lower, although consistent, at ∼5%. The thicker cell wall of the Gram-positive Firmicutes compared to the Gram-negative Deltaproteobacteria in conjunction with the gDNA extraction method may be a reason for the difference in extraction efficiency (29, 33). The endogenous levels of *hgcA*^+^ Deltaproteobacteria and Firmicutes were determined to be ∼1.43 × 10^5^ and ∼4.55 × 10^5^ cell copies per dry gram sediment, respectively, after accounting for the average extraction efficiency of all spiked samples (Fig. 4).

As for the Archaea, the results were less clear. From melt curve analysis (data not shown) and gel electrophoresis (Fig. 3) of the sediment amplicon, two dominant products were observed. One product was at the correct position, and the second product was much smaller, thereby artificially inflating the *hgcA* abundance. A more modern take on PCR, epicPCR (emulsion, paired isolation, and concatenation PCR) (34), may be a solution to the problem observed with the Archaea; however, it is a less routine and more involved method. In short, this approach effectively links phylogeny (via 16S rRNA genes) to the gene of interest (i.e., *hcgA*) followed by sequencing. Therefore, the incorrectly assigned sequences could be excluded from downstream calculations. However, typical PCR amplification limitations would still apply and should still be considered. The inability to recover the methanogen spike in this experiment may have been the result of the specific choice of an organism with poor gDNA extraction efficiency and/or qPCR recovery.

### Conclusion.

We have successfully designed and optimized a universal, broad-range degenerate primer set for *hgcAB*. This primer set amplifies an ∼950-bp product, which was sequence verified from gDNA isolated from 26 *hgcAB*^*+*^ pure cultures. The diversity of organisms amplified by our method is much greater than other previously published approaches. The *hgcAB*^*+*^ broad-range primer set serves as an inexpensive diagnostic tool for identifying the presence of Hg methylation potential in environmental samples. We also designed and optimized three clade-specific sets of degenerate qPCR primers that amplify *hgcA*. The novel clade-specific qPCR primer sets will allow for more accurate and robust quantification individually of the three dominant Hg-methylating groups of microorganisms: Deltaproteobacteria, Firmicutes, and methanogenic Archaea that have different abilities to methylate mercury (4). While they do not amplify from every known Hg-methylating organism, the majority of tested organisms responded to the appropriate primers.

In order to quantitatively apply the *hgcA* qPCR primer sets in natural samples, some *a priori* understanding of community structure may be needed, as well as an evaluation of *hgcA* recovery, by clade or organism, from the sample of interest. During method development, we recommended that 16S rRNA gene diversity profiling and/or *hgcAB* amplification with high-throughput sequencing be employed along with qPCR *hgcA* quantification to more fully evaluate the approach, including the potential for an off-target signal. A more quantitative understanding of gDNA extraction and amplification efficiencies for *hgcAB*^+^ organisms should allow for the development of correction factors to estimate *in situ hgcA* densities.

Development of these primer sets for *hgcA* and *hgcAB* is an important step toward understanding the distribution of Hg-methylating organisms in nature. Adaptation of these primer sets for gene expression in nature should be possible, allowing direct measurement of methylation potential. This new information will lead in turn to the development of robust and sensitive hydrobiogeochemical models of Hg methylation in the environment.

## Supplementary Material

Supplemental material

## References

[1] Amos HM, Jacob DJ, Streets DG, Sunderland EM 2013 Legacy impacts of all-time anthropogenic emissions on the global mercury cycle. Global Biogeochem Cycles 27:410–421. doi:10.1002/gbc.20040.

[2] Reinfelder JR, Fisher NS, Luoma SN, Nichols JW, Wang WX 1998 Trace element trophic transfer in aquatic organisms: a critique of the kinetic model approach. Sci Total Environ 219:117–135. doi:10.1016/S0048-9697(98)00225-3. 9802246

[3] Hsu-Kim H, Kucharzyk KH, Zhang T, Deshusses MA 2013 Mechanisms regulating mercury bioavailability for methylating microorganisms in the aquatic environment: a critical review. Environ Sci Technol 47:2441–2456. doi:10.1021/es304370g. 23384298

[4] Gilmour CC, Podar M, Bullock AL, Graham AM, Brown SD, Somenahally AC, Johs A, Hurt RA Jr, Bailey KL, Elias DA 2013 Mercury methylation by novel microorganisms from new environments. Environ Sci Technol 47:11810–11820. doi:10.1021/es403075t. 24024607

[5] Podar M, Gilmour CC, Brandt CC, Soren A, Brown SD, Crable BR, Palumbo AV, Somenahally AC, Elias DA 2015 Global prevalence and distribution of genes and microorganisms involved in mercury methylation. Sci Adv 1:e1500675. doi:10.1126/sciadv.1500675. 26601305 PMC4646819

[6] Jeremiason JD, Engstrom DR, Swain EB, Nater EA, Johnson BM, Almendinger JE, Monson BA, Kolka RK 2006 Sulfate addition increases methylmercury production in an experimental wetland. Environ Sci Technol 40:3800–3806. doi:10.1021/es0524144. 16830545

[7] Mitchell CPJ, Branfireun BA, Kolka RK 2008 Assessing sulfate and carbon controls on net methylmercury production in peatlands: an *in situ* mesocosm approach. Appl Geochem 23:503–518. doi:10.1016/j.apgeochem.2007.12.020.

[8] Marvin-DiPasquale M, Windham-Myers L, Agee JL, Kakouros E, Kieu le H, Fleck JA, Alpers CN, Stricker CA 2014 Methylmercury production in sediment from agricultural and nonagricultural wetlands in the Yolo Bypass, California, USA. Sci Total Environ 484:288–299. doi:10.1016/j.scitotenv.2013.09.098. 24188689

[9] Gilmour CC, Elias DA, Kucken AM, Brown SD, Palumbo AV, Schadt CW, Wall JD 2011 Sulfate-reducing bacterium Desulfovibrio desulfuricans ND132 as a model for understanding bacterial mercury methylation. Appl Environ Microbiol 77:3938–3951. doi:10.1128/AEM.02993-10. 21515733 PMC3131654

[10] Hamelin S, Amyot M, Barkay T, Wang YP, Planas D 2011 Methanogens: principal methylators of mercury in lake periphyton. Environ Sci Technol 45:7693–7700. doi:10.1021/es2010072. 21875053

[11] Yu R-Q, Reinfelder JR, Hines ME, Barkay T 2013 Mercury methylation by the methanogen Methanospirillum hungatei. Appl Environ Microbiol 79:6325–6330. doi:10.1128/AEM.01556-13. 23934484 PMC3811210

[12] Fleming EJ, Mack EE, Green PG, Nelson DC 2006 Mercury methylation from unexpected sources: molybdate-inhibited freshwater sediments and an iron-reducing bacterium. Appl Environ Microbiol 72:457–464. doi:10.1128/AEM.72.1.457-464.2006. 16391078 PMC1352261

[13] Yu R-Q, Flanders JR, Mack EE, Turner R, Mirza MB, Barkay T 2012 Contribution of coexisting sulfate and iron-reducing bacteria to methylmercury production in freshwater river sediments. Environ Sci Technol 46:2684–2691. doi:10.1021/es2033718. 22148328

[14] Parks JM, Johs A, Podar M, Bridou R, Hurt RA Jr, Smith SD, Tomanicek SJ, Qian Y, Brown SD, Brandt CC, Palumbo AV, Smith JC, Wall JD, Elias DA, Liang L 2013 The genetic basis for bacterial mercury methylation. Science 339:1332–1335. doi:10.1126/science.1230667. 23393089

[15] Schaefer JK, Kronberg RM, Morel FMM, Skyllberg U 2014 Detection of a key Hg methylation gene, *hgcA*, in wetland soils. Environ Microbiol Rep 6:441–447. doi:10.1111/1758-2229.12136. 25646534

[16] Liu Y, Yu R, Zheng Y, He J 2014 Analysis of the microbial community structure by monitoring an Hg methylation gene (*hgcA*) in paddy soils along an Hg gradient. Appl Environ Microbiol 80:2874–2879. doi:10.1128/AEM.04225-13. 24584244 PMC3993304

[17] Bae H, Dierberg FE, Ogram A 2014 Syntrophs dominate sequences associated with the mercury methylation-related gene *hgcA* in the water conservation areas of the Florida Everglades. Appl Environ Microbiol 80:6517–6526. doi:10.1128/AEM.01666-14. 25107983 PMC4178645

[18] Bravo AG, Loizeau JL, Dranguet P, Makri S, Bjorn E, Ungureanu VG, Slaveykova VI, Cosio C 2016 Persistent Hg contamination and occurrence of Hg-methylating transcript (*hgcA*) downstream of a chlor-alkali plant in the Olt River (Romania). Environ Sci Pollut Res Int 23:10529–10541. doi:10.1007/s11356-015-5906-4. 26662302

[19] Hintelmann H, Evans RD 1997 Application of stable isotopes in environmental tracer studies: measurement of monomethylmercury (CH_3_Hg^+^) by isotope dilution ICP-MS and detection of species transformation. Fresenius J Anal Chem 358:378–385. doi:10.1007/s002160050433.

[20] Edgar RC 2004 MUSCLE: a multiple sequence alignment method with reduced time and space complexity. BMC Bioinformatics 5:113. doi:10.1186/1471-2105-5-113. 15318951 PMC517706

[21] Larsson A 2014 AliView: a fast and lightweight alignment viewer and editor for large datasets. Bioinformatics 30:3276–3278. doi:10.1093/bioinformatics/btu531. 25095880 PMC4221126

[22] Larkin MA, Blackshields G, Brown NP, Chenna R, McGettigan PA, McWilliam H, Valentin F, Wallace IM, Wilm A, Lopez R, Thompson JD, Gibson TJ, Higgins DG 2007 Clustal W and Clustal X version 2.0. Bioinformatics 23:2947–2948. doi:10.1093/bioinformatics/btm404. 17846036

[23] Bustin SA, Benes V, Garson JA, Hellemans J, Huggett J, Kubista M, Mueller R, Nolan T, Pfaffl MW, Shipley GL, Vandesompele J, Wittwer CT 2009 The MIQE guidelines: minimum information for publication of quantitative real-time PCR experiments. Clin Chem 55:611–622. doi:10.1373/clinchem.2008.112797. 19246619

[24] Bustin SA 2010 Why the need for qPCR publication guidelines? The case for MIQE. Methods 50:217–226. doi:10.1016/j.ymeth.2009.12.006. 20025972

[25] Pfaffl MW 2001 A new mathematical model for relative quantification in real-time RT-PCR. Nucleic Acids Res 29:e45. doi:10.1093/nar/29.9.e45. 11328886 PMC55695

[26] Hellemans J, Mortier G, De Paepe A, Speleman F, Vandesompele J 2007 qBase relative quantification framework and software for management and automated analysis of real-time quantitative PCR data. Genome Biol 8:R19. doi:10.1186/gb-2007-8-2-r19. 17291332 PMC1852402

[27] Vandesompele J, De Preter K, Pattyn F, Poppe B, Van Roy N, De Paepe A, Speleman F 2002 Accurate normalization of real-time quantitative RT-PCR data by geometric averaging of multiple internal control genes. Genome Biol 3:RESEARCH0034. 12184808 10.1186/gb-2002-3-7-research0034 PMC126239

[28] Southworth GR, Greeley MS, Peterson MJ, Lowe K, Ketelle RH 2010 Sources of mercury to East Fork Poplar Creek downstream from the Y-12 national security complex: inventories and export rates. Oak Ridge National Laboratory, Oak Ridge, TN.

[29] Hurt RA, Robeson MS, Shakya M, Moberly JG, Vishnivetskaya TA, Gu B, Elias DA 2014 Improved yield of high molecular weight DNA coincides with increased microbial diversity access from iron oxide cemented subsurface clay environments. PLoS One 9:e102826. doi:10.1371/journal.pone.0102826. 25033199 PMC4102596

[30] Hurt RA, Brown SD, Podar M, Palumbo AV, Elias DA 2012 Sequencing intractable DNA to close microbial genomes. PLoS One 7:e41295. doi:10.1371/journal.pone.0041295. 22859974 PMC3409199

[31] Robb FT, Maeder DL, Brown JR, DiRuggiero J, Stump MD, Yeh RK, Weiss RB, Dunn DM 2001 Genomic sequence of hyperthermophile, Pyrococcus furiosus: implications for physiology and enzymology. Methods Enzymol 330:134–157. doi:10.1016/S0076-6879(01)30372-5. 11210495

[32] Brown SD, Wall JD, Kucken AM, Gilmour CC, Podar M, Brandt CC, Teshima H, Detter JC, Han CS, Land ML, Lucas S, Han J, Pennacchio L, Nolan M, Pitluck S, Woyke T, Goodwin L, Palumbo AV, Elias DA 2011 Genome sequence of the mercury-methylating and pleomorphic Desulfovibrio africanus strain Walvis Bay. J Bacteriol 193:4037–4038. doi:10.1128/JB.05223-11. 21642452 PMC3147520

[33] Vishnivetskaya TA, Layton AC, Lau MC, Chauhan A, Cheng KR, Meyers AJ, Murphy JR, Rogers AW, Saarunya GS, Williams DE, Pfiffner SM, Biggerstaff JP, Stackhouse BT, Phelps TJ, Whyte L, Sayler GS, Onstott TC 2014 Commercial DNA extraction kits impact observed microbial community composition in permafrost samples. FEMS Microbiol Ecol 87:217–230. doi:10.1111/1574-6941.12219. 24102625

[34] Spencer SJ, Tamminen MV, Preheim SP, Guo MT, Briggs AW, Brito IL, D AW, Pitkanen LK, Vigneault F, Virta MP, Alm EJ 2016 Massively parallel sequencing of single cells by epicPCR links functional genes with phylogenetic markers. ISME J 10:427–436. doi:10.1038/ismej.2015.124. 26394010 PMC4737934

