# Supplementary material for: Development and Validation of Broad-Range Qualitative and Clade-Specific Quantitative Molecular Probes for Assessing Mercury Methylation in the Environment
Source: Appl Environ Microbiol. 2016 Sep 16;82(19):6068–78. doi: 10.1128/AEM.01271-16 (PMC5038027; doi:10.1128/AEM.01271-16)
Supplement: Supplemental material [file supp_82_19_6068__index.html]

Supplemental material 

# Development and Validation of Broad-Range Qualitative and Clade-Specific Quantitative Molecular Probes for Assessing Mercury Methylation in the Environment

## Supplemental material

- Supplemental file 1 -

  Supplemental text: primer testing, *bgcAB* primer design, selected cloning, additional modifications to culture media for select strains; polymerase and temperature comparison for broad-range PCR protocol (Fig. S1); gel electrophoresis of qPCR products of isolate gDNA with *Deltaproteobacteria* primer set for 31 strains (Fig. S2); degenerate versus specific primer sets tested with *Dv. desulfuricans* ND132 (Fig. S3); gel electrophoresis of qPCR products of isolate gDNA with methanogenic *Archaea* primer set for 31 strains (Fig. S4); gel electrophoresis of qPCR products of isolate gDNA with *Firmicutes* primer set for 31 strains (Fig. S5); legends to tables in Data Set S1.

  PDF, 977K
- Supplemental file 2 -

  Data Set S1: strain information and expected broad-range PCR product size (Table S1); complete list of oligonucleotides (primers) tested (Table S2); primer alignment and results for broad-range *hgcAB* PCR and clade-specific *hgcA* qPCR (Table S3); primer alignment for each primer set for all *hgcAB*+ microorganisms (Table S4); amplification protocols (Table S5); sensitivity of clade-specific primers (Table S6); sand versus sediment qPCR results (Table S7).

  XLSX, 129K
