# Supplementary material for: Development and Validation of Broad-Range Qualitative and Clade-Specific Quantitative Molecular Probes for Assessing Mercury Methylation in the Environment
Source: Appl Environ Microbiol. 2016 Sep 16;82(19):6068–78. doi: 10.1128/AEM.01271-16 (PMC5038027; doi:10.1128/AEM.01271-16)
Supplement: Supplemental material [file AEM.01271-16_zam999117415so1.pdf]

Supplemental Text:

Primer Testing: As a first determination of primer efficacy, qualitative and quantitative primers were tested on cell pellets before being tested on extracted DNA. Pelleted stationary phase cultures were added to a 25 µl mixture of TaqRED and primer through the use of a sterile inoculating loop first swirled through the pellet and then into the PCR mixture. After running the PCR as specified within this report, the PCR products were checked on agarose gels to look for positive bands. Primers that showed positive results were then optimized for DNA concentration.

hgcA Primer Design: Many different primer combinations were tested prior to the selection of the ones described and are listed in Table S2. In order to create the different primers, *hgcAB* sequences were aligned by clade using Aliview [1] or Clustal X2 [2] and primers created visually by inspecting for conserved sequences spanning upstream or downstream of the cap-helix site. Potential primers were first tested *in silico* using Primer-BLAST (http://www.ncbi.nlm.nih.gov/tools/primer-blast/) against the different *hgcAB*$^+$ organisms sequenced. Primer combinations showing positive results on Primer-BLAST were then subjected to laboratory screening to test for positive bands on agarose gels.

hgcA Primer Design: During this study, as previously reported [3], we observed that the *Deltaproteobacteria* typically have a higher GC-content as compared to the other two clades. Of the 47 *hgcAB*$^+$ *Deltaproteobacteria* studied here, the average genome GC-content was approximately 57% and consequently the primers designed had higher melting temperatures (Tm's) for amplification (Table S4). To determine the optimized annealing temperature, multiple experiments were performed with representative strains from each of the clades at 55 to70°C. Although at lower temperatures the *hgcA*$^+$ *Deltaproteobacteria* amplified well, so too did strains from the other clades. Non-specific products were observed for non-*Deltaproteobacteria* by gel electrophoresis and melt curve analysis.

Above 66°C the efficiency of these primers, even for several of the *Deltaproteobacteria*, was poor. Therefore, in order to limit amplification of non-*Deltaproteobacteria* gDNA and still maintain reasonable amplification of the correct single product, 65°C annealing/extension step was concluded to satisfy this constraint.

Next, the concentration for each of the primers was tested with a gradient range of 100-2000 nM final primer concentration. Below 200 nM primer efficiency was typically <50%, and above 500 nM non-specific amplification was observed. Therefore, 250 nM was determined to be acceptable for the *Deltaproteobacteria* qPCR primers. The annealing/extension time was also modified from 30 seconds, which allowed for sufficient amplification, to 20 seconds since the former allowed for non-*Deltaproteobacteria* amplification and 10 seconds was too short as observed by a decreased efficiency in *Deltaproteobacteria* amplification. Typically, 40 cycles were sufficient for qPCR analysis, with 30 cycles being the minimum. Because the goal was to amplify *Deltaproteobacteria* specifically and no other strains whose sequence may be similar, 30 cycles were chosen. Some of the non-*Deltaproteobacteria* strains did still amplify a single product at the correct band size, but did not produce a signal until after 30 cycles with 2.5×10^6 copies as compared with the *Deltaproteobacteria* at 16-20 cycles confirming specificity (data not shown).

Of the 22 *Firmicutes* examined in this study, the average GC-content was lower than the *Deltaproteobacteria,* at 45% and consequently the primers had lower Tm's (Table S4). Initial examination of the *Firmicutes* sequences revealed two unique groupings. One included the sulfate- and sulfite-reducing *Firmicutes,* where conserved sequences allowed for straightforward primer design. The second group, including the fermenting *Firmicutes*, was much more diverse in sequence and significantly complicated the strategy for primer design. In an effort to optimize a single primer set for all *Firmicutes*, the results as seen by gel electrophoresis and melt curve analysis showed

undesirable and/or multiple products, or the degeneracy of the primer set was outside of what we found to be tractable; >500-fold degeneracy resulted in unacceptable levels of non-specific. Therefore, in optimizing primers for *Firmicutes*, only the more conserved sulfate- or sulfite-reducing *Firmicutes* were considered. Similar procedures were performed on the *Firmicutes* as was performed on the *Deltaproteobacteria* in an effort to optimize the protocols (data not shown).

Of the 13 methanogenic *Archaea* examined, the average GC-content was 51%, placing this clade between the other two. The sequences for these strains are also quite diverse, which lead to the increased degeneracy and consequently more difficulty in separating archaea signal from non-archaea strains. At 2.5 x 10^6 copies of template gDNA amplification was observed by qPCR, as seen by a fluorescent signal, for several non-methanogenic *Archaea*, although the product was typically not *hgcA* as determined by melt curve analysis and gel electrophoresis as described later. An increase in the extension temperature from 55°C to 60°C led to amplification of a noticeably larger product for *Ml. tindarius*. Additionally, a decrease to 45°C increased non-specific amplification across most strains tested (data not shown). Taken together, the anneal temperature and consequently the extension temperature was restricted to between 50°C and 55°C for optimization. In regard to optimization with respect to altered primer concentration, an increase above 250 nM typically lead to an increase in non-specific amplification while a decrease to 200 nM or less lead to unacceptable loss in amplification efficiency.

A primer set was also designed to include only the 13 *hgcAB*$^+$ methanogenic *Archaea* sequences currently available with the expectation that a degeneracy of 13-fold for both the forward and reverse primers separately would limit non-specific amplification as compared with the set previously described. Similar results for the sequence specific primers were observed as for those previously described and were no longer pursued (data not shown). A decrease in degeneracy of the

primer sequences was typically followed by a concomitant loss of amplification and primer efficiency for many of the methanogenic *Archaea* strains for other primers tested, effectively forcing the methanogenic primers to being more degenerate.

Selected cloning: To further examine the smaller band (~50 bp) observed for some of the archaea strains with the archaea qPCR primer set we used the Archaea *hgcA* qPCR primers to amplify from gDNA isolated from *Ml. tindarius* as well as two environmental samples from anaerobic sediments. We used the protocol as stated in the methods, but extended the cycles from 30 to 40. The PCR product was subjected to electrophoresis. The region spanning just below the band for the desired product to just above the band for the primers was excised (this region contained one blurred band at approximately 50 bp). The DNA from the excised gel was extracted with the Wizard® SV Gel Clean-Up Kit (Promega). We then used the TA Cloning® Kit (Life Technologies) to generate clones of the purified PCR products. Twenty individual colonies were picked and screened for inserts using primers located on the recombinant plasmid (M13fwd and M13rev). Five of the 20 screened colonies from *Ml. tindarius* and 21 of the 40 screened colonies from the environmental samples had inserts and additional subcultures were extracted using the GeneJET™ Plasmid Miniprep Kit (Thermo Fisher Scientific, Waltham, MA). The purified plasmids were sequenced using the Sanger protocol at University of Tennessee Molecular Biology Resource Facility.

The sequenced clones from *Ml. tindarius* returned reads of only *hgcA* (112 bp), indicating that cloning of the small product was unsuccessful. The sequenced clones from the environmental sample returned reads of 45-86 bp. The 45 bp product was the *hgcA* forward primer followed by the *hgcA* reverse primer. The longer reads contained the *hgcA* forward primer, followed by DNA that did not blast to any known gene, and ended with the *hgcA* reverse primer. We conclude that the

degenerate primers in combination with the low annealing temperature generate non-specific amplification. However, our optimization experiments showed that a lower degeneracy or higher annealing temperature results in unacceptably low levels of amplification.

Additional modifications to culture media for select strains.

Desulfovibrio inopinatus: DSM medium 193 was altered by increasing NaCl to 10g/L, adding yeast extract (YE, 0.5 g/L), substituting MOPS buffer (15 mM) for carbonate buffer, and substituting 100 mM cysteine for sulfide.

Ethanoligenens harbinense: DSM medium 1057 was altered by using the trace element solution from DSM Medium 633 instead of 318.

Methanospirillum hungatei: The recipe used was an acetate (5mM)/formate (30mM) medium provide by P. Browne. Salts were KH_2PO_4 (0.1 g/L), $MgSO_4$ x $7H_2O$ (0.2 g/L), $CaCl_2$ x $2H_2O$ (0.03 g/L), NH_4Cl (1 g/L), and KCl (0.1 g/L). It contained 1g/L YE, 5 ml/L Tanner's Trace metals and 10 ml/L vitamin solution from DSM medium 141. The medium was buffered with 45 mM bicarbonate adjusted to pH 7.5, and reduced with 100 µM sulfide and 500 µM cysteine. Cultures were grown at 34°C under 80:20 H_2:CO_2 at 20 psi.

Methanofollis liminatans: Medium was modified from Zellner and Jargon 1997 by adding 1g/L YE, omitting peptone and sulfate, and adding sodium acetate (2g/L) and sodium formate (2g/L).

Methanocorpusculum bavaricum: DSM medium 279 was altered by omitting sludge fluid and adding clarified rumen fluid (100 mg/L).

Methanosphaerula palustris E1-9c: DSM medium 1094 was altered by omitting Solutions B and C, increasing sodium acetate to 4mM and Coenzyme M to 0.6mM, and by adding DSM medium 141

trace element solution (1mL/L). The medium was reduced with 0.5mM titanium (III) nitrilotriacetate and 500uM cysteine.

Methanocella paludicola SANAE: Medium was modified from Sekiguchi 2000 by adding 0.1g/L YE, 0.1g/L sodium acetate and 1mL/L vitamin solution and omitting Fe(III)nitrilotriacetate.

References (Supplemental Text):

1. **Larsson, A.** 2014. AliView: a fast and lightweight alignment viewer and editor for large datasets. Bioinformatics **30:**3276-8.
2. **Larkin, M. A., G. Blackshields, N. P. Brown, R. Chenna, P. A. McGettigan, H. McWilliam, F. Valentin, I. M. Wallace, A. Wilm, R. Lopez, J. D. Thompson, T. J. Gibson, and D. G. Higgins.** 2007. Clustal W and Clustal X version 2.0. Bioinformatics **23:**2947-8.
3. **Lightfield, J., N. R. Fram, and B. Ely.** 2011. Across bacterial phyla, distantly-related genomes with similar genomic GC content have similar patterns of amino acid usage. *PLoS ONE*, **6**(3):e17677.

References (Supplemental Tables):

Bae H, Dierberg FE, Ogram A. 2014. Syntrophs dominate sequences associated with the mercury methylation-related gene *hgcA* in the water conservation areas of the Florida everglades. Appl Environ Microbiol 80:6517-6526.

Friedrich, M., N. Springer, W. Ludwig, B. Schink. 1996. Phylogenetic positions of *Desulfofustis glycolicus* gen nov, sp nov, and *Syntrophobotulus glycolicus* gen nov, sp nov, two new strict anaerobes growing with glycolic acid. Int J Syst Bacteriol **46**:1065-1069.

Gilmour CC, Elias DA, Kucken AM, Brown SD, Palumbo AV, Schadt CW, Wall JD. 2011. Sulfate-Reducing Bacterium *Desulfovibrio desulfuricans* ND132 as a Model for Understanding Bacterial Mercury Methylation. Applied and Environmental Microbiology 77:3938-3951.

Gilmour C, Podar M, Bullock AL, Graham AM, Brown SD, Somenahally AC, Johs A, Hurt RA, Jr., Bailey KL, Elias DA. 2013. Mercury Methylation by Novel Microorganisms from New Environments. Environ Sci Technol 47:11810-11820.

Graham AM, Bullock AL, Maizel AC, Elias EA, Gilmour CC. 2012. Detailed Assessment of the Kinetics of Hg-Cell Association, Hg Methylation, and Methylmercury Degradation in Several *Desulfovibrio* Species. Applied and Environmental Microbiology 78(20):7337-7346.

Hai, T., D. Lange, R. Rabus, A. Steinbuchel. 2004. Polyhydroxyalkanoate (PHA) accumulation in sulfate-reducing bacteria and identification of a class III PHA synthase (PhaEC) in *Desulfococcus multivorans*. Appl Environ Microbiol **70**:4440-4448.

King JK, Kostka JE, Frischer ME, Saunders FM. 2000. Sulfate-reducing bacteria methylate mercury at variable rates in pure culture and in marine sediments. Applied and Environmental Microbiology 66(6):2430-2437.

Liu Y, Yu R, Zheng Y, He J. 2014. Analysis of the microbial community structure by monitoring an Hg methylation gene (*hgcA*) in paddy soils along an Hg gradient Appl Environ Microbiol 80:2874-2879.

Moberly JG, Brown SD, Kucken AM, Gilmour CC, Brandt CC, Palumbo AV, Wall JD, Elias DA. 2012. Role of morphological growth state and gene expression in *Desulfovibrio africanus* strain Walvis Bay mercury methylation. Environ Sci Technol 46(3):7926-32.

Parks JM, Johs A, Podar M, Bridou R, Hurt RA, Jr., Smith SD, Tomanicek SJ, Qian Y, Brown SD, Brandt CC, Palumbo AV, Smith JC, Wall JD, Elias DA, Liang L. 2013. The genetic basis for bacterial mercury methylation. Science 339:1332-1335.

Podar M, Gilmour CC, Brandt CC, Soren A, Brown SD, Crable BR, Palumbo AV, Somenahally AC, Elias DA. 2015. Global prevalence and distribution of genes and microorganisms involved in mercury methylation. Sci Adv 1:e1500675.

Schaefer JK, Kronberg RM, Morel FMM, Skyllberg U. 2014. Detection of a key Hg methylation gene, *hgcA*, in wetland soils. Environ Microbiol Rep 6:441-447.

Yu R-Q, Reinfelder JR, Hines ME, Barkay T. 2013. Mercury methylation by the methanogen *Methanospirillum hungatei*. Applied and Environmental Microbiology Published ahead of print August 9, 2013, doi: 10.1128/AEM.01556-13.

Supplemental Figures:

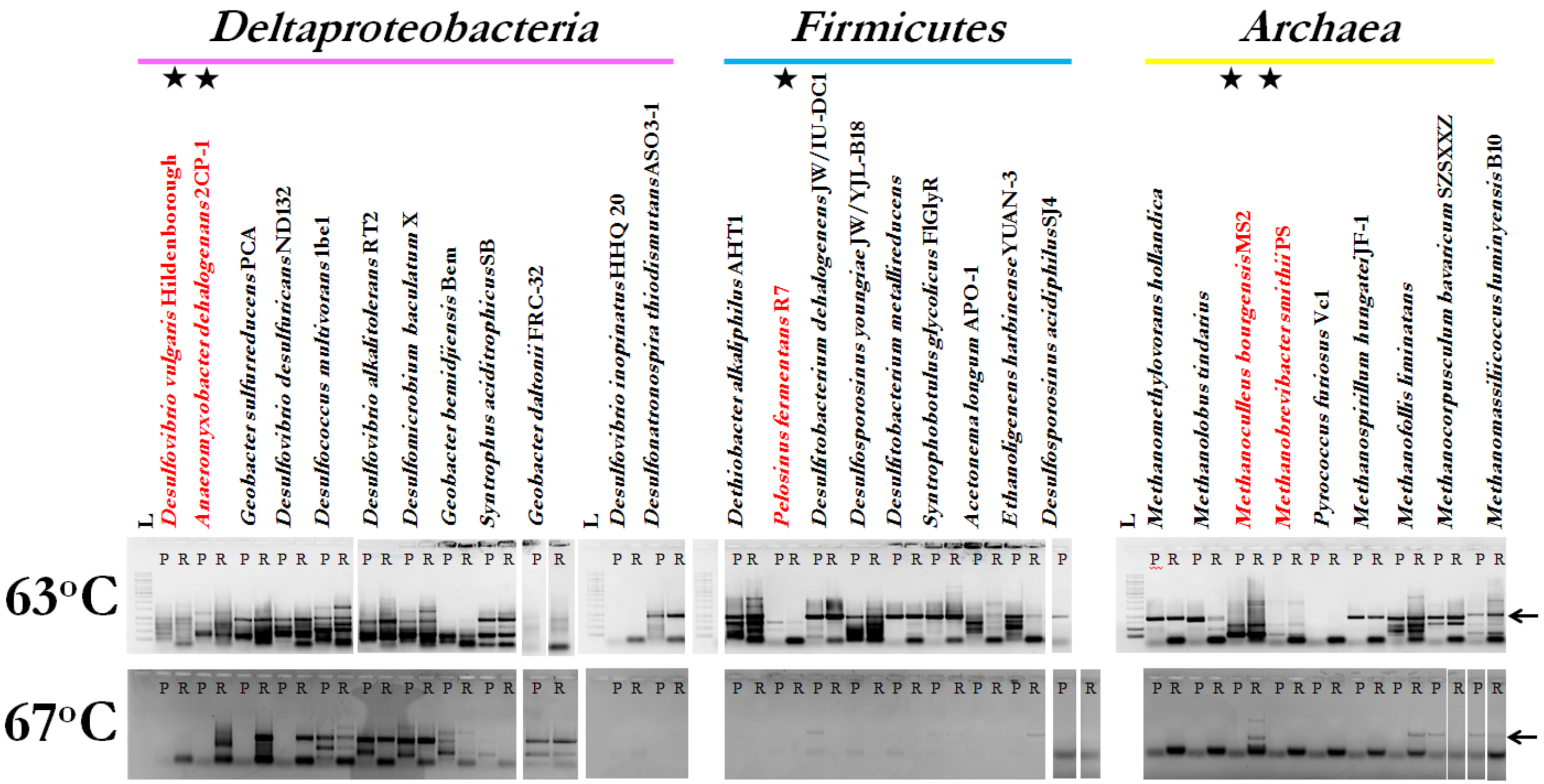

Figure S1: Polymerase and temperature comparison for broad-range PCR protocol. PCR performed on 30 isolate gDNA with either Invitrogen™ Platinum™ *Taq* (P) or Apex™ TaqRED (R) DNA polymerase with an annealing temperature at 63°C or 67°C, respectively. A star and red text denotes a microorganism that does not encode *hgcAB* and should not generate a product, while the rest are *hgcAB*$^{+}$ microorganisms. Arrow denotes expected band size position (~950 bp). Ladder (L) is 1kb Plus from Thermo Fisher Scientific.

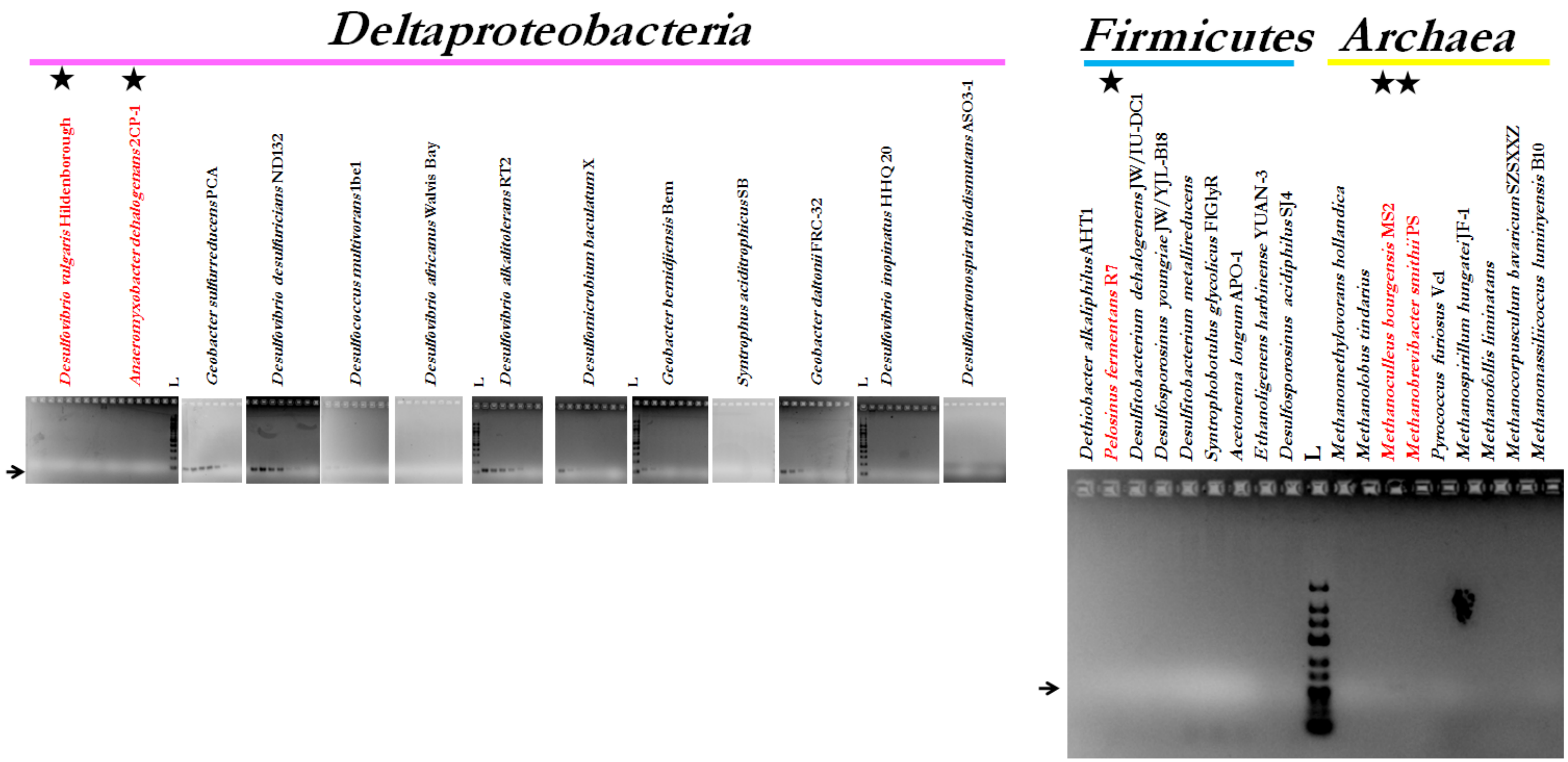

Figure S2: Gel electrophoresis of qPCR of isolate gDNA with *Deltaproteobacteria* primer set for 31 strains. For each *Deltaproteobacteria* strain and for each dilution one of each of the triplicates was passed through an agarose gel. Dilutions: seven 4-fold serial dilutions, 2.5 x 10^6 to 1.5 x 10^2 copies per reaction. For the *Firmicutes* and methanogenic *Archaea* strains the 2.5 x 10^6 sample was loaded only. The ladder (L) was 1kb Plus DNA Ladder for the left image and O'GeneRuler Low Range DNA Ladder for the right image. Black arrow points to the expected size (107 bp). Organisms in red are starred and denote microorganisms that do not encode for *hgcAB* and should not generate a product, while the rest are *hgcAB*$^+$ microorganisms. A product should only be observed for *hgcAB*$^+$ *Deltaproteobacteria*.

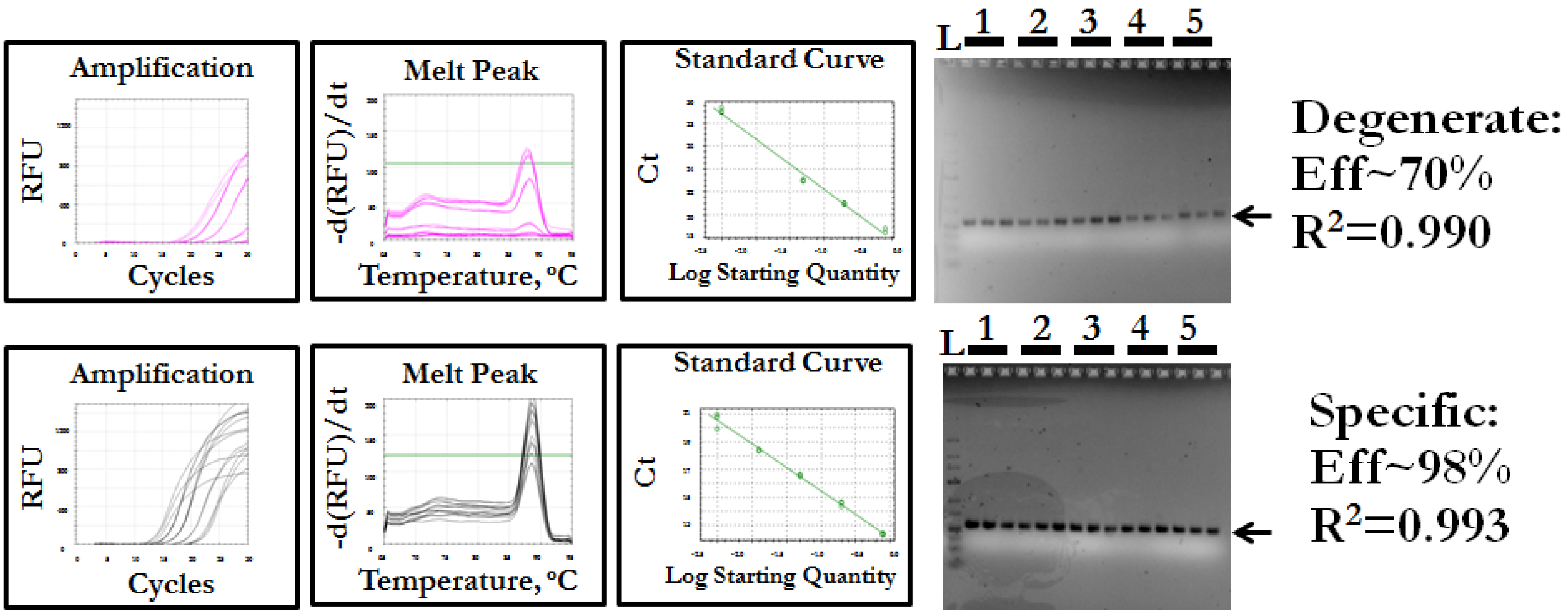

Figure S3: Degenerate versus specific primer sets tested with *Dv. desulfuricans* ND132. Parallel qPCR experiments were performed with gDNA with either (TOP) the currently designed *Deltaproteobacteria* primer set (ORNL-Delta-HgcA-F with ORNL-Delta-HgcA-R) or with (BOTTOM) specific primers (ORNL-D-ND132-F with ORNL-D-ND132-R) with the exact sequence for *Dv. desulfuricans* ND132. To calculate primer efficiency, technical replicates for each gDNA concentration (1, 2.5 x 10^6; 2, 6.25 x 10^5; 3, 1.56 x 10^5; 4, 3.91 x 10^4; and 5, 9.76 x 10^3 copies per reaction) were analyzed by qPCR and the products were passed through an agarose gel. The DNA ladder (L) was O'GeneRuler Low Range DNA Ladder. The expected product as indicated by an arrow was 107 bp.

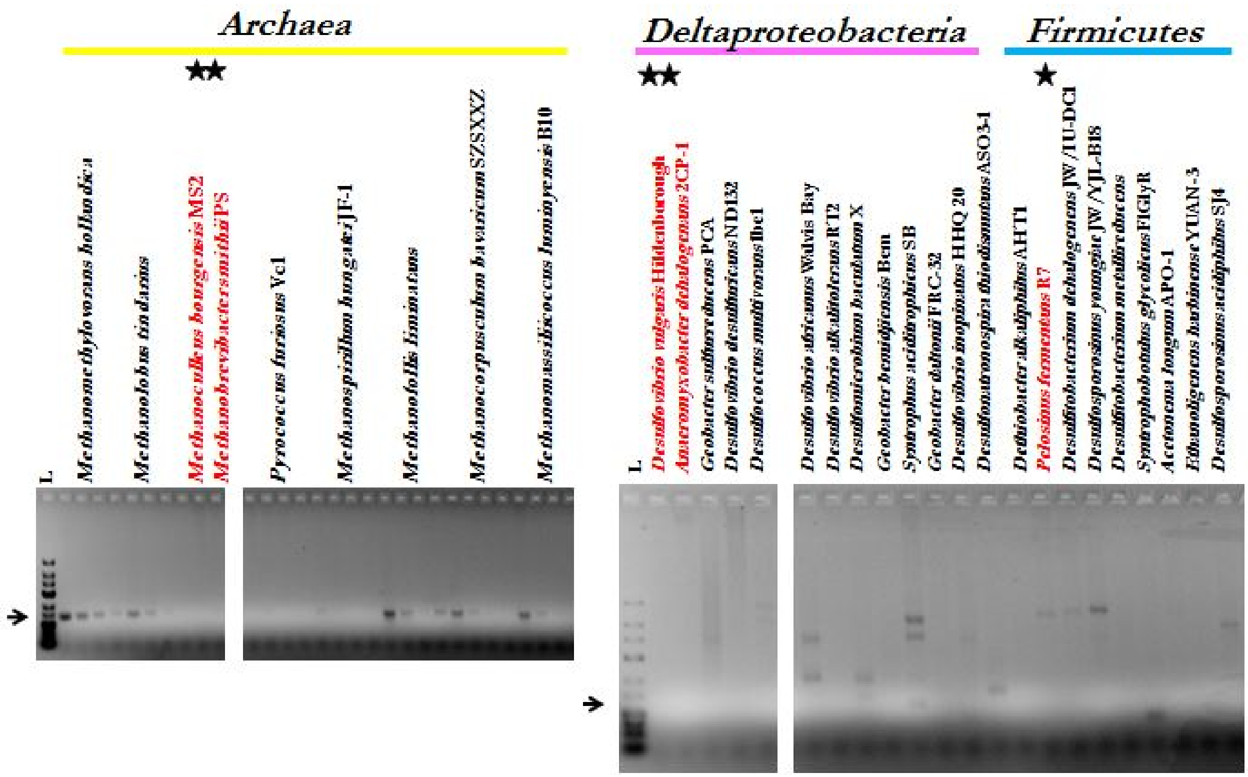

Figure S4: Gel electrophoresis of qPCR products of isolate gDNA with methanogenic *Archaea* primer set for 31 strains. Five additional cycles, 35 in total, were added to emphasize non-specific amplification that occurred and the need to limit the cycle number to 30. For each methanogenic *Archaea* strain and for each dilution one of each of the triplicate qPCR was passed through an agarose gel. Dilutions: seven 4-fold serial dilutions, 2.5×10^6 to 1.5×10^2 copies per reaction. For the two *hgcAB*$^-$ methanogenic *Archaea*, *Deltaproteobacteria* and *Firmicutes* strains only the 2.5×10^6 sample was loaded. The ladder (L) was O'GeneRuler Low Range DNA Ladder. Black arrow points to the expected size (125 bp). Organisms in red are starred and denote microorganisms that do not encode for *hgcAB* and should not generate a product, while the rest are *hgcAB*$^+$ microorganisms. A product should only be observed for *hgcAB*$^+$ methanogenic *Archaea*. At 35 cycles, many of the non-*Archaea* strains had a band(s) following this protocol. However, only *Desulfonatronospira thiodismutans* appeared to be at the correct size. Because of the non-specific amplification observed after 35 cycles, 30 cycles were chosen to limit undesired product amplification.

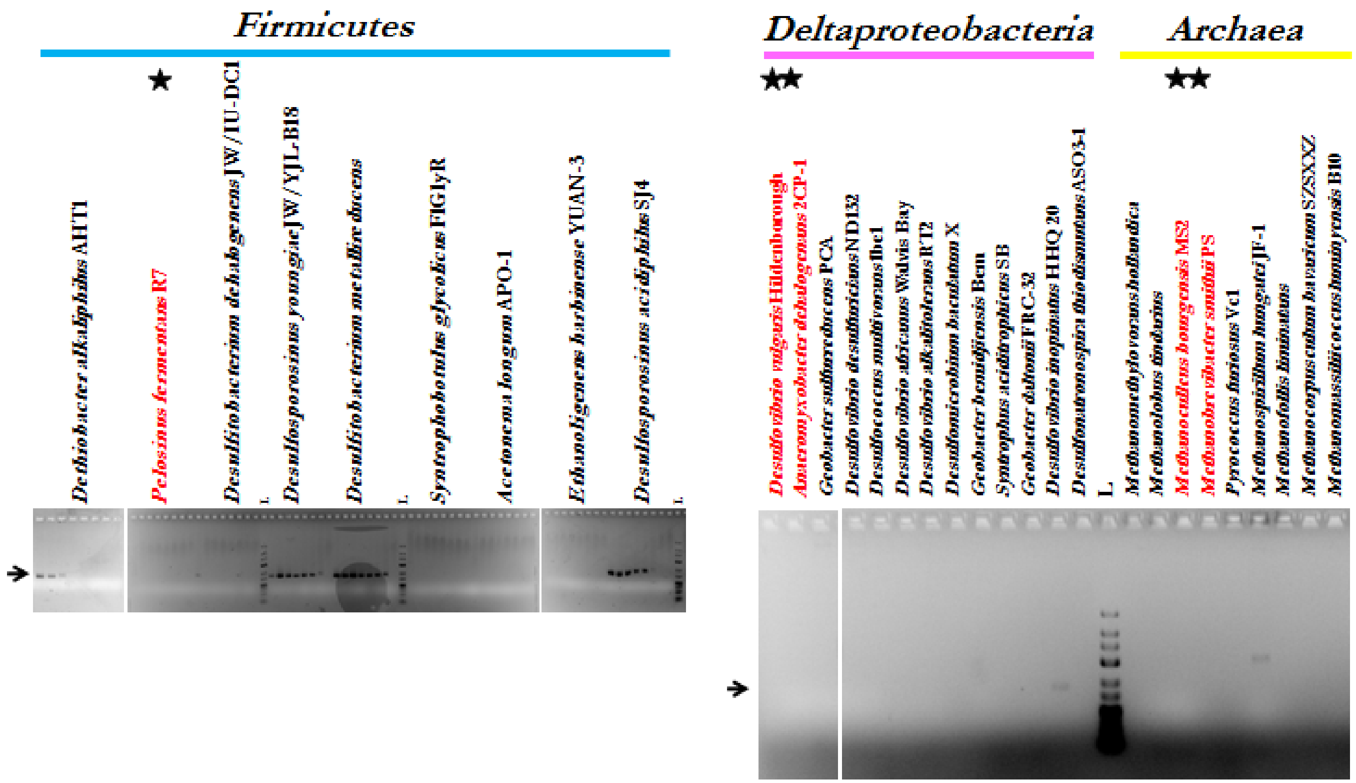

Figure S5: Gel electrophoresis of qPCR products of isolate gDNA with *Firmicutes* primer set for 31 strains. For each *Firmicutes* strain and for each dilution one of each of the triplicate qPCR was passed through an agarose gel. Dilutions: seven 4-fold serial dilutions, 2.5 x 10^6 to 1.5 x 10^2 copies per reaction. For the *Deltaproteobacteria* and methanogenic *Archaea* strains only the 2.5 x 10^6 sample was loaded. The ladder was O'GeneRuler Low Range DNA Ladder. Black arrow points to the expected size (167 bp). Organisms in red are starred and denote microorganisms that do not encode for *hgcAB* and should not generate a product, while the rest are *hgcAB*$^+$ microorganisms. A product should only be observed for sulfate-reducing *hgcAB*$^+$ *Firmicutes*.

Supplemental Tables:

Table S1: Strain information and expected broad-range PCR product size.
Refer to Excel sheet for complete table.

Table S2: Complete list of oligonucleotides (primers) tested.
Refer to Excel sheet for complete table.

Table S3: Primer alignment and results for broad-range *hgcAB* PCR and clade-specific *hgcA* qPCR.
Refer to Excel sheet for complete table.

Table S4: Primer alignment for each primer set for all *hgcAB*$^+$ microorganisms.
Refer to Excel sheet for complete table.

Table S5: Amplification Protocols.
Refer to Excel sheet for complete table.

Table S6: Sensitivity of clade-specific primers.
Refer to Excel sheet for complete table.

Table S7: Sand versus sediment qPCR results.
Refer to Excel sheet for complete table.
